# An open source web application for the surveillance and prevention of the impacts on public health of extreme meteorological events: the SUPREME system

**DOI:** 10.1186/1476-072X-10-39

**Published:** 2011-05-25

**Authors:** Steve Toutant, Pierre Gosselin, Diane Bélanger, Ray Bustinza, Sonia Rivest

**Affiliations:** 1 Institut national de santé publique du Québec, 945 Wolfe, Quebec (Quebec), G1V 5B3, Canada; 2 Centre de recherche du CHUQ, bureau 600, 2875 boul. Laurier, Quebec (Quebec), G1V 2M2, Canada; 3 Institut national de la recherche scientifique (INRS-ETE), 490 de la Couronne, Quebec (Quebec), G1K 9A9, Canada; 4 Intelli3 Inc., 2750 rue Einstein, bureau 130, Quebec (Quebec) G1P 4R, Canada

## Abstract

**Background:**

Every year, many deaths or health problems are directly linked to heat waves. Consequently, numerous jurisdictions around the world have developed intervention plans that are employed during extreme heat events; beyond their emergency sections, these plans generally include preventive measures to be implemented each year. Over the last five years, local and regional information systems have been implemented in a few Canadian cities for surveillance purposes. However, until recently, no such systems existed at the provincial level. In the context of the Government of Quebec's 2006-2012 Action Plan on Climate Change, a real-time integrated system for the surveillance and monitoring of extreme heat events has been implemented on a provincial level. The system is a component of a broader approach that would also monitor the public health impacts of all types of extreme meteorological events.

**Results:**

After conducting a detailed needs analysis, the Quebec National Institute for Public Health developed and implemented an integrated web application leveraging open source software for the real-time Surveillance and Prevention of the impacts of Extreme Meteorological Events on public health, called the SUPREME system. Its first field use involved heat waves. This decision-support system is based on open source software and is composed of four modules: (1) data acquisition and integration, (2) risk analysis and alerts, (3), cartographic application, and (4) information dissemination - climate change and health portal. The system is available to health specialists through a secure web information portal and provides access to weather forecasts, historic and real-time indicators (including deaths and hospital admissions), alerts and various cartographic data used for conducting prevention activities and launching emergency measures.

**Conclusions:**

The SUPREME system was implemented and used during the summer of 2010. It served as an important decision-making tool during the July 2010 heat wave in the province of Quebec, Canada. Planned improvements for 2011 include the integration of data related to other risk factors for other extreme events to the system. The next steps will be to provide access to the application to other groups of specialists that are involved in the prevention, monitoring, or analysis of extreme meteorological events and their effects on community health and well-being.

## Background

In 2003, many European countries, France and Italy particularly, were hit by a record-breaking heat wave. It is well known that heat waves lead to higher morbidity and mortality, predominantly among the elderly and socially isolated persons with pre-existing cardiovascular, respiratory and some other chronic diseases. In fact, estimates of additional deaths recorded that summer in Europe range from 20 000 to upwards of 70 000 [1,2]. Heat-related deaths are expected to grow as a consequence of projected further increases in the frequency, intensity, and duration of heat waves due to climate change, as well as increases in average summer temperatures [3]. For example, a ten-year study carried out by the PHEWE project [4] in 15 European cities, estimated a 2% increase in mortality in northern cities and a 3% increase in southern cities for every one-degree Celsius increase in apparent temperature above the city threshold level. A similar 20-year study done in Quebec (latitude comparable to northern European cities) showed a similar mortality increase of about 2% per degree Celsius [5]. Apparent temperature is a measure of relative discomfort due to combined heat and high humidity.

Since 2004, a few local and regional information systems have been implemented in the province of Quebec in order to facilitate the monitoring of extreme heat events and mortality and morbidity rates. However, there was no such system available at the provincial scale that would allow for the surveillance of the impacts of heat waves on the health of the entire at-risk population and serve as an emergency decision-support system. Without a comprehensive view of the impacts of extreme weather conditions on a population's health, it remains difficult to predict an increase in the mortality and morbidity rates in the different regions in the advent of a heat wave, and to set-up the required preventive and appropriate curative actions.

In order to close this gap, and in the context of the Government of Quebec's 2006-2012 Action Plan on Climate Change (APCC) [6], the Ministry of Health and Social Services (MHSS) mandated the Quebec National Institute of Public Health (QNIPH) to develop a real-time, integrated system for the surveillance and monitoring of extreme heat events, with prevention as well as emergency preparedness in mind. Over time, the initial concept has evolved into a common web application for use in all types of extreme meteorological events (including their hydrological and geological consequences), with a first application being its heat component.

The heat component, developed in 2009-2010, was operational as of June 1^st^, 2010. This paper first describes the methods used to develop the objectives and requirements of the system. The results, namely the software architecture and the capabilities of the system, are presented, followed by a discussion of how the system was used during the July 2010 heat wave in Quebec. Finally, the paper will briefly explain the improvements that are currently under development and planned to be integrated in the SUPREME system in 2011.

## Methods

The needs analysis is described first, and then the general and specific objectives of the system are presented. Criteria for software framework selection are then listed.

### The needs analysis

The QNIPH developed an online survey in order to understand the actual processes used in each of the 18 health regions of Quebec for the surveillance of extreme heat events, and to garner the required improvements to the processes. The survey was based on an official heat wave intervention and preparedness guide developed in 2005-2006 [7] and was sent to all potential end users in the regional public health directorates. It comprised more than 60 questions grouped into four sections. The first section aimed at identifying the processes that were in use in each health region within the province. The second section aimed at identifying the perceived gaps in these processes in order to develop the strategic and operational objectives. The objective of section 3 was to identify the groups, within the population, that were at risk in their respective regions, in the opinion of the end users. Finally, section 4 helped to identify and characterize the key actors responsible for the surveillance and monitoring of extreme heat events at the local level. An open question, at the end of the survey, allowed for the gathering of additional information and comments useful in the context of the development of the new system. The survey was reviewed by an expert and researchers in the field of climate change and extreme heat, before being submitted to all the representatives within each health region. Of the 22 persons targeted by the survey, 16 actually participated and their participation covered 17 of the 18 health regions (one of the participants being responsible for the heat questions in two regions).

Examples of survey questions are presented in table 1.

**Table 1:** Examples of questions for the needs analysis

Section	Example of question
Current processes	While at work, are you being notified of extreme heat alerts, and if yes, by which organization(s) and mean(s)?

Gaps	Which aspects of your intervention plan in case of extreme heat should be improved?

Risk characterization	Have risks been localized geographically? Have the groups of population that are at risk been identified and localized geographically?

Key actors	Including yourself, how many people in your organization are involved in extreme heat-related issues? Do you have quick and easy access to sanitary data?

The results of the survey were compiled and presented to the Heat committee of the Quebec Coordinating Group on Environmental Health in February 2009. The survey results, as well as the outcomes of the meeting, were used to identify common needs in terms of the information and the capabilities required in the system to be implemented at the provincial level and hence to define the specifications of the future system.

### Objectives of the system for its heat component

The main objective of the project was to design, develop, and implement an integrated system for the surveillance and monitoring of extreme heat events for each of the health regions within the province of Quebec.

The specific objectives were to provide the following information to all provincial public health actors in real-time:

- a complete meteorological picture (actual conditions, alerts and forecasts); the population's health status based on relevant indicators; air quality data; specific forecasts for extreme heat events (heat events causing an increase in the mortality and/or morbidity rate(s) as defined by health experts);

- surveillance indicators in order to evaluate, *a posteriori*, the impacts of extreme heat events on the population's health at regional and provincial scales;

The identified required datasets included: current meteorological conditions; short- and mid-term meteorological forecasts (3 days); historical meteorological indicators; the location of the groups of population that are at risk (according to various indicators); the location of urban heat islands; air quality monitoring; the relevant health and health care data; the location of features relevant for field intervention such as health centers, emergency centers, cooling centers, schools, nursing homes and senior's residences, green spaces and parks, pools and beaches, and other public buildings. Other data suggested by users for future inclusion included detailed information about municipal emergency plans.

### Criteria for software selection

The selected software and code libraries to be included in the architecture had to present the following characteristics: well documented and well supported by large user and developer communities; high interoperability that conforms to international standards; multiplatform; easy to deploy; and low acquisition cost and maintenance fees.

## Results

This section describes the design and implementation processes, as well as the architecture and capabilities, of the SUPREME system.

### Development of the SUPREME system

**Figure 1:**
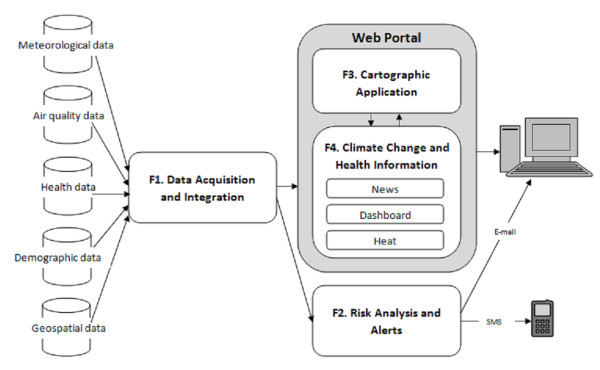
**The general architecture of the SUPREME system**. The system is composed of four functions: (1) the data acquisition and integration function, (2) the risk analysis and alerts function, (3) the cartographic application function, and (4) the climate change and health portal function.

The general architecture of the SUPREME system is presented in figure 1.

The system is based on an open source software framework. The software and the libraries that are part of this framework are all from recognized and well supported open source projects. This ensures a degree of maturity and a continuity of the solution. For example, the geospatial software components used in Function 3 are mostly from Open Source Geospatial Foundation (OSGEO) [8] projects and the Web Map Services (WMS) created conform to the Open Geospatial Consortium (OGC) standards.

The choice of open-source solutions was ultimately determined by budgetary considerations and is based on the geographic information cooperation principles of the Government of Quebec.

Most of the systems developed at the QNIPH aim at fulfilling specific needs. For example, the monitoring of the evolution of a pandemic (H1N1), supporting an epidemiologist during an epidemiologic study, helping in the selection of samples for a new health survey, etc. Once these projects are completed, the supporting systems rarely evolve, however, they must remain accessible for many years despite the absence of a recurrent budget for their maintenance. In this context, it is difficult to demonstrate the return on investment for expenses related to the acquisition and annual maintenance of proprietary software.

Because any software or API, proprietary or open-source, only seldom meets the web cartographic dissemination and spatial analysis needs in an "out of the box" manner, the use of free APIs allowed for investing in development time instead of acquisition and maintenance fees. This reduced technological dependency and allowed for the development of a lighter application that only included the required capabilities.

The community aspect of the use of open source software was also appealing because the software is the product of a vision that is built on the sharing of knowledge and programming code, and the mutual support of its users. This vision is underpinned by mutual aid and sharing values, values that are also shared by an organization working in the public health field.

**Figure 2:**
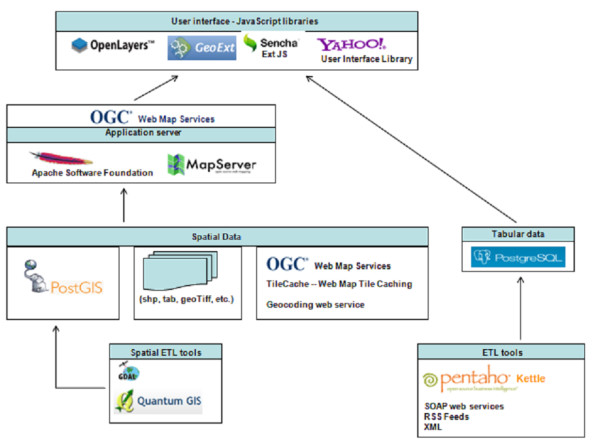
**The software architecture of the SUPREME system**. The architecture is based on open source software components from recognized open source projects.

The software framework used to develop the system is presented in Figure 2. The system comprises four functions: F1 - The data acquisition and integration function; F2 - The risk analysis and alerts function; F3 - The cartographic application function; F4 - The climate change and health information function. These functions will be described in more detail in the following sections.

### Function 1. Data acquisition and integration

The objective of this function is to ensure that the data required for the surveillance and analysis activities are obtained, integrated, updated and ready to use in the other functions of the system. This function comprises the following six (6) tasks: urban heat islands, vulnerability indicators, meteorological data and public warnings, health data, air quality, and geospatial data.

#### Urban heat islands

A methodology has been developed at QNIPH in order to extract thermal information from Landsat satellite images, taking into account a number of variables of importance for public health [9]. The classification of these images allows for determining the warmest zones for densely inhabited areas. An image has been produced for all the health regions where the determination of urban heat islands is relevant. These images are read by the function and available for display in the cartographic application (see *Function 3. Cartographic information *below).

#### Vulnerability indicators

A number of indicators have been selected by end users, from the list presented in a QNIPH publication by Tairou *et al. *[10], based on a systematic review of relevant risks factors and health determinants in heat waves. These indicators are listed in Table 2.

**Table 2:** Vulnerability indicators included in the SUPREME system (heat)

Indicator	Source
Regional deprivation index - 2006	QNIPH - based on census data

Population density	Census data

Age	Census data

Housing conditions	Census data

Landed immigrants since 2001	Census data

Foreign language population	Census data

Dissemination areas inside heat islands	QNIPH - based on census geographic data

These indicators are available at the dissemination area level [11], which is the smallest stable census area in Canada (average of 700 people). Using the functions of the portal, a user can parameterize each of the indicators, for example by selecting only a subset of age groups (e.g. ≥75 years), or a category of housing conditions, and launch a query that will return the list of all the dissemination areas that correspond to the selected multi-criteria analysis (e.g. areas with proportion of deprived households ≥10%, located within an urban heat island, with proportion of immigrants ≥50%), thus allowing better targeting and use of adequate language for further communication. The query result can be visualized on a map using the cartographic application. Hence, each user can establish his/her own vulnerability criteria, and these can be customized according to the region being analyzed, thus taking into account the regional socio-demographic specificities (for instance, few immigrants or older neighbourhood or higher income) and allowing for setting regional intervention priorities and adapting tools and messages.

#### Meteorological data and public warnings

Environment Canada, the Canadian federal organization responsible for providing weather and environmental forecasts to the Canadian population, has implemented RSS feeds that contain, among other information, the meteorological warnings in effect in each city. Every hour, the SUPREME system reads the relevant RSS feeds and the warnings are processed and transferred to Function 2 for dissemination (see *Function 2. Risk analysis and alerts *below).

The QNIPH, along with a research partner, the National Institute of Scientific Research (NISR), has established temperature thresholds above which there is a risk of high mortality rates due to extreme heat. Every hour, the system obtains the 72- hour forecasts from Environment Canada (in the form of XML files), for each relevant forecast region. The minimum and maximum temperatures and the humidity index (called Humidex), for the next 24, 48 and 72 hours, are used to calculate 3-day weighted temperatures and a 3-day weighted humidity index, according to a weight method described in [12]. This information is then transferred to Functions 2 and 4 for dissemination.

The system also makes a daily compilation of historic meteorological data coming from selected meteorological stations, one station per health region. The historic meteorological data are also used in Function 4 for the production of graphics presenting the variations of temperature along with other indicators (see *Function 4. Climate change and health information *below).

#### Health data

The system currently obtains health data regarding deaths, hospitalizations, emergency admissions, ambulance trips, and calls to health information centers. Two data sources are used to obtain daily information: the daily record of the situation at emergency departments and hospitals, and the health information daily report. Some other information of potential use for emergency preparedness is made available in the system, such as the location of medical services (clinics, hospitals, etc.), cooling centers, green spaces and parks, pools and beaches.

#### Air quality

A real-time image of the Air Quality Index is obtained from the Quebec Ministry of Sustainable Development, Environment and Parks, and is directly available in the Climate change and Health Information portal of the system.

#### Geospatial data

The system obtains geospatial data from many sources of the Government of Quebec (and its agencies) and in various formats. Raster data are stored on the server in their native format. Vector data are integrated in a PostgreSQL/PostGIS database. Some data sources are accessed using the WMS protocol [13], and the images obtained are ready to be displayed in the cartographic application. Currently, the application accesses heterogeneous data formats such as Tilecache [14], PostGIS, Shapefile, MapInfo, WMS, text, and geocoding.

### Function 2. Risk analysis and alerts

The objectives of this function are to redirect the high heat and humidity warnings as issued by Environment Canada, and to disseminate the warnings associated with an increased risk of mortality as calculated using the thresholds proposed by the QNIPH [10]. Each of these objectives corresponds to a specific task.

#### High heat and humidity warnings from Environment Canada

Environment Canada issues a warning when the temperature is above 30°C, and the humidity index is equal to, or above, 40; this warning is the same all over the province. The current task redirects the warnings to the involved public health actors, using email and/or short message service (SMS).

#### High mortality risk warnings from QNIPH

**Figure 3:**
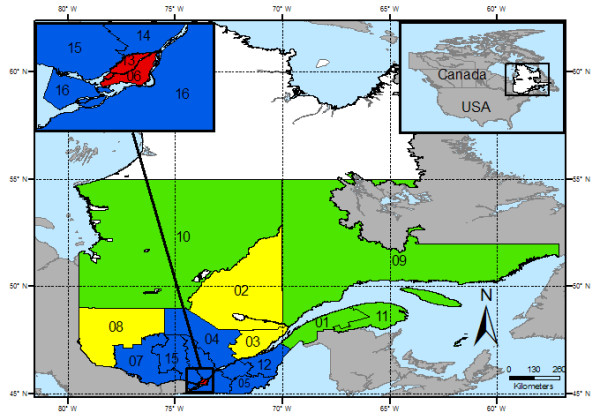
**Health regions of southern Quebec grouped into homogenous climatic classes**. The map shows the four climatic classes for the province of Quebec.

The weighted 3-day forecasts (cf. *Meteorological data and public warnings *section above) are compared to thresholds established by the QNIPH. If the thresholds are reached or exceeded, a message is transmitted, by email and/or SMS to the involved public health officers. A weighted forecast over 3 days was deemed optimal to identify high risk periods for excessive mortality in a previous study [12]. This led to significant differences in recommended thresholds across the province. For instance, the Montreal area threshold (#6 in red zone in Figure 3) was identified as a maximum of 33°C, a minimum of 20°C and a humidity index of 40, with daily weights of 40-40-20%. Quebec City, which is only 250 km North-East (#3 in yellow zone) but cooled by the St. Lawrence River and estuarine tides, would reach the same risk zone at 31°C and 16°C with the same weights, and a humidity index of 37 [12].

### Function 3. Cartographic application

This function is available through the SUPREME system web portal. The objective of this function is to create a cartographic interface allowing for the visualization of geographic data related to the risks, the protection factors and the vulnerable areas. The cartographic interface also offers spatial analysis capabilities for deriving new information. This function comprises five tasks: display of maps, cartographic navigation tools, manipulation of geospatial data layers, geocoding, and spatial analysis. The first two tasks are standard cartographic application tools and will not be described in details. The three other tasks allow for selecting and enriching existing geospatial data.

#### Manipulation of geospatial data layers

All the available data layers are presented to users. Some of the layers are displayed by default on the map. Users can choose to display or hide certain data layers, and can also add new external WMS layers to the map.

#### Geocoding

This task allows for placing a marker on the map at specific geographic coordinates and for obtaining socio-demographic data related to the selected location. The coordinates can be calculated/displayed using an address, a city name or a postal code; using latitude and longitude; the actual center of the map extent; or a mouse click on a location on the map.

#### Spatial analysis

Linked to the dissemination areas layer is a query that allows for displaying vulnerable areas corresponding to the combination of particular user-selected indicator values.

**Figure 4:**
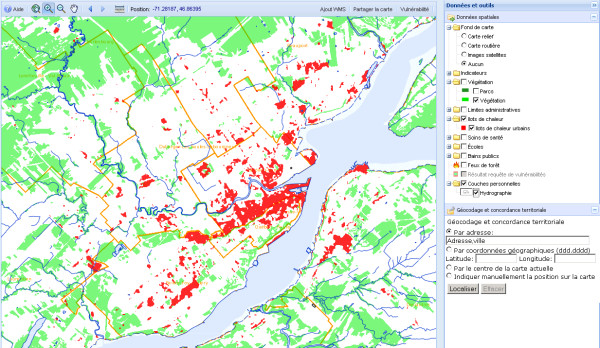
**The cartographic interface of the SUPREME system (heat)**. The interface is composed of the map display zone, the cartographic navigation tools (top left), the data layers and the geocoding tools (on the right), and the menu (top right) allowing for adding WMS layers, sharing the map, and displaying custom vulnerable areas.

Figure 4 shows an example of cartographic display. Users can save their cartographic context (i.e. the current map extent and the displayed layers) in order to reuse it at a later time and/or share the map with other users.

### Function 4. Climate change and health information

In addition to the cartographic application described in the previous section, the system web portal comprises a section for the dissemination of relevant information in the form of tables, charts, and textual information. This function comprises three tasks: news, dashboard and heat information. Each of these tasks is presented in a different tab in the portal interface.

#### News

This section of the portal presents real-time news sources about the environment and climate changes in general, through a set of RSS feeds.

#### Dashboard

**Figure 5:**
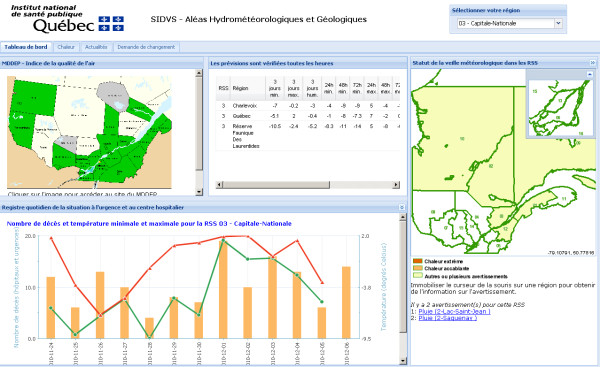
**The dashboard section of the SUPREME system (heat)**. The dashboard is composed of the air quality map (top left), a table of the meteorological forecasts and the weighted forecasts (top center), a map of the current warnings from Environment Canada (on the right), and a chart showing the minimum and maximum temperatures as well as the number of deaths, per day, for the last two weeks.

This section presents tables and charts of the meteorological forecasts, mortality data, air quality data and current meteorological warnings. These data are described in details in section *Function 1. Data acquisition and integration *above. The tables and charts are updated many times a day, seven days a week. Figure 5 presents a view of the dashboard section of the portal.

#### Health information

**Figure 6:**
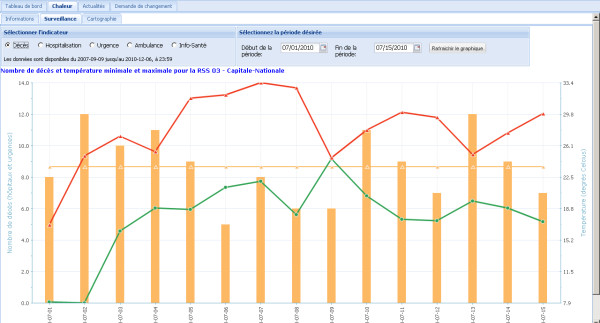
**An example of chart available in the surveillance section of the SUPREME heat portal**. This chart presents the daily number of deaths, as well as the minimum and maximum temperatures. The chart spans 15 days by default.

This section comprises three subsections: information, surveillance and cartography. The first subsection presents information on the system, various relevant documents, and hyperlinks to interesting web sites. The second subsection, surveillance, contains a chart of the daily number of deaths, new hospitalizations, emergency admissions, ambulance trips, calls to health information centers, as well as the minimum and maximum temperatures. The data sources used are described in details in section *Function 1. Data acquisition and integration *above. The user can select the desired health indicator(s) and can also modify the period covered by the chart. The third subsection contains a hyperlink to the cartographic application described in the previous section. Figure 6 presents an example of chart available in the surveillance subsection.

## Discussion

If the proof of the pudding is in the eating, as the saying goes, then what's better than a severe heat wave to put a new system to test? Our web application described above was launched on June 1^st^, 2010, and followed shortly by a heat wave.

### The July 2010 heat wave in Quebec

In Canada, the summer of 2010 was one of the hottest summers according to national records of temperature [15]. The worst heat waves in 50 years hit the southern parts of Ontario and Quebec. From July 5^th ^to July 9^th^, in most southern regions of Quebec, maximum temperatures reached 30°C and up to 35°C, minimum temperatures stayed well above 20°C, and the humidity index was very high, above 40 [16]. The SUPREME system supplied very useful information to the various health actors during that period. This information was used for evaluating the situation in the field, in real-time, and in a continuous mode.

On July 5^th^, the system began transmitting warnings to actors from the MHSS and 12 affected health regions. For 10 of these regions, the warnings related to extreme heat (with high risk of excess mortality) and the regions deployed their action plan at the alert or the full mobilization stages. Moreover, due to the scale of the heat wave, telephone conferences were organized by the MHSS with the health regions and the QNIPH in order to coordinate and harmonize the measures against the potential impacts of the heat wave on the population's health. Close contacts were also maintained with emergency preparedness officers at all levels.

A preliminary analysis of the data supplied by the SUPREME system indicated various impacts on population health in certain health regions concerned by the warnings. Compared to the average number of deaths (all causes) in the period of July 1^st ^to July 15^th ^of the preceding two years, the variation in mortality was +30,1% for all affected areas in the province [17]. A comparison using the two-week period prior to the 2010 heat wave yielded similar levels, around +24%. The comparison of the daily total number of hospitalizations, emergency visits and ambulance trips showed similar increases in most affected health regions. The increase in the number of calls to health information centers was very significant and was proportional to the increase in the daily maximum temperature in most health regions having received extreme heat warnings, going from 1 or 2 calls per day on heat-related problems before the wave to more than 100 per day, for most regions.

As a point of comparison, mortality data for the Montreal island health region during two previous shorter heat waves (each episode lasting 2 days in1987 and 1994) yielded an increase of daily mortality well over 100% each time [18], while this event peaked at 28,9% for a 5-day duration with higher temperatures [17]. This is a good argument for the efficacy of improved emergency preparedness and for supporting tools such as the SUPREME system, probably aided by the progression of air conditioning availability in nursing homes and similar venues.

### The SUPREME system compared to other similar systems

In Canada, systems similar to the SUPREME system exist at a regional or at a municipal scale, for example in Montreal and Toronto. There is no such system at a provincial scale. The situation is similar in the United-States, where, for example, the cities of Chicago and Philadelphia each have their extreme heat systems but state-wide or regional systems are non-existent.

To our knowledge, no other system offers a dynamic cartographic application showing the urban heat islands and having tools for identifying the vulnerable areas using a combination of numerous user-selected and user-controlled indicators. Furthermore, no other system created cartographic Web services that can be reused within other systems.

### Evaluation of the system

After its first months of operation, the usefulness of the system has been evaluated by users in order to improve its capabilities and usability [19], and also to prepare for future integration of new features. Some 86% of end users found the system very or somewhat useful and 97% found it easy to use (high or medium ease of use). About one third of users completed surveillance reports based on the information found in the application.

The system was accessed mainly during the extreme heat events in order for the users to obtain a health portrait of their region and of the neighbouring regions, and to visualize the status of the extreme heat warnings in the province of Quebec. Of the 14% of users who found the system less useful, some of them mentioned that extreme heat events are not an issue in their region. A few others mentioned that a communication network or an information system addressing the extreme heat problem was already implemented in their region.

The users mentioned that the cartographic application will be particularly useful for prevention and for preparing for a heat season, for example by helping to identify the population located in heat islands or other vulnerable areas.

During the summer of 2010, approximately 60 users from the health network used the system for their extreme heat surveillance needs. Also, in 2009 and 2010, the cartographic application was made available to the general public in order for the municipalities and the companies to propose projects for adapting to, or preventing the formation of, heat islands. In this context more that 4000 people accessed the application.

When compared to previous methods and processes used in the health regions, the system presents many improvements. It allows for a uniform access to relevant health data, air quality data, and meteorological data through a single entry point. It manages the heat warnings and disseminates valid information to the relevant actors, in a timely fashion. It also allows for easy cartographic visualization of risks, vulnerability, and protection factors, not to mention easy post-event reporting.

### Future of the system

Future improvements to the system include the integration of new indicators and datasets, as well as an access for other specialized user communities (e.g. emergency preparedness specialists, urban planners). For 2011, other meteorological and geological risks factors are under development for inclusion into the application: intense cold, storms, floods, and landslides (as determined by a literature review). A user group has been constituted for each of the new risk factors to be included in order to specify data needs and the functions to be developed into the system. Close cooperation with other government departments has allowed this development to take place very swiftly.

The current clients of the system are from the MHSS, the QNIPH, the public health directorates and agencies in each health region, local health centers, and cities. The system is only accessible through the health telecommunications network, a secure network designated for health-related specialists. In relation to the new risk factors to be included, the system will be opened to the associated interested user communities and available on the Internet.

## Conclusion

This paper presents an integrated and operational web application leveraging open source software for the real-time Surveillance and Prevention of Extreme Meteorological Events impacts on public health, called the SUPREME system, and its first field use for heat waves. This includes its context, architecture, and capabilities, examples of outputs, as well as a discussion of how the system was used during the July 2010 heat wave in the southern regions of the province of Quebec, Canada. Planned improvements to the system are also briefly presented. The SUPREME system (heat component) has been developed by the Quebec National Institute of Public Health (QNIPH) in response to the requirements of the Government of Quebec's 2006-2012 Action Plan on Climate Change (APCC).

The SUPREME system became an important decision-support tool during the July 2010 heat wave in Quebec for the actors responsible for heat-related health issues within the various health regions and at the Ministry of Health and Social Services (MHSS), particularly in the regions that did not have prior access to such type of system. The system represented the single common source for all the relevant heat-related information and allowed for real-time delivery of shared and valid information to all involved actors.

Thanks to the information supplied by the SUPREME system, to appropriate surveillance, to the measures deployed by the health regions representatives, and to the coordination efforts of the MHSS, it is possible to assess that the effects of the heat wave on the population's health have been controlled and even reduced compared to past experiences.

The architecture of the SUPREME system is based on recognized and well-supported open source software and libraries. It is an excellent example of integration of these technologies within the public health field. We believe our approach can be useful for other jurisdictions planning to implement or improve similar systems, either in Canada or elsewhere. Within the QNIPH, the framework of the system is currently being reused for several other projects in infectious diseases.

## List of abbreviations

APCC: Action Plan on Climate Change; MHSS: Ministry of Health and Social Services; NISR: National Institute of Scientific Research; OGC: Open Geospatial Consortium; OSGEO: Open Source Geospatial Foundation; PHEWE: Assessment and Prevention of acute Health Effects of Weather conditions in Europe; QNIPH: Quebec National Institute of Public Health; RSS: Really Simple Syndication; SHP: Shapefile; SMS: Short Message Service; SUPREME system: integrated web application for Surveillance and Prevention of Extreme Meteorological Events impacts on public health; TIFF: Tagged Image File Format; WMS: Web Map Server; XML: Extensible Markup Language.

## Competing interests

The authors declare that they have no competing interests.

## Authors' contributions

ST was in charge of the development and implementation of the system. He revised the paper. PG contributed to the development and implementation of the system, contributed to the writing and revised the paper. DB contributed to the development and implementation of the system and revised the paper. RB contributed to the implementation and evaluation of the system and revised the paper. SR was responsible for the writing of the paper. All authors read and approved the final manuscript.
